# Genetic engineering of parthenocarpic tomato plants using transient *SlIAA9* knockdown by novel tissue-specific promoters

**DOI:** 10.1038/s41598-019-55400-7

**Published:** 2019-12-11

**Authors:** Ji-Seong Kim, Kentaro Ezura, Jeongeun Lee, Tohru Ariizumi, Hiroshi Ezura

**Affiliations:** 1 Faculty of Life and Environmental Sciences, University of Tsukuba, Tennodai 1-1-1 Tsukuba, Ibaraki, 305-8572 Japan; 2 Tsukuba Plant Innovation Research Center, University of Tsukuba, Tennodai 1-1-1, Tsukuba, Ibaraki, 305-8572 Japan

**Keywords:** Molecular engineering in plants, Molecular engineering in plants

## Abstract

Parthenocarpy is the development of an ovary into a seedless fruit without pollination. The ubiquitous downregulation of *SlIAA9* induces not only parthenocarpic fruit formation but also an abnormal vegetative phenotype. To make parthenocarpic transgenic tomato plants without unwanted phenotypes, we found two genes, namely, *Solyc03g007780* and *Solyc02g067760*, expressed in ovary tissue but not in vegetative tissues. *Solyc03g007780* was expressed in developing ovaries and anthers. *Solyc02g067760* mRNA was detected in whole-flower tissues. The promoters of *Solyc03g007780* (Psol80) and *Solyc02g067760* (Psol60) predominantly induced the expression of genes in the ovule, placenta, endocarp and pollen and in whole-flower tissues, respectively. *Psol80/60-SlIAA9i* lines, created for *SlIAA9-*RNA interference controlled by two promoters, successfully formed parthenocarpic fruits without pleiotropic effects in vegetative tissues. Downregulation of *SlIAA9*, responsible for parthenocarpic fruit formation, was observed in ovules rather than ovaries in the *Psol80/60-SlIAA9i* lines. Although the weight of parthenocarpic fruits of the *Psol80/60-SlIAA9i* lines was lower than the weight of pollinated fruits of the wild type (WT), the parthenocarpic fruits presented redder and more saturated colors and higher levels of total soluble solids and titratable acidity than the WT fruits.

## Introduction

Due to the importance of tomato fruit as a food, tomato fruit set (the initiation of development of an ovary into fruit) and ripening have been studied for several decades^1–4^. Successful fertilization is essential for fruit set and development followed by the formation of seeds, which are a source of many hormones for cell division and subsequent cell expansion^4,5^. Although cultivated tomatoes have an appropriate flower structure for self-pollination (i.e., the tomato flower has both a pistil and stamens that are similar in length), physical vibration is still needed to complete successful pollination. Additionally, despite the presence of this shaking, failed fruit set can occur under low light or high temperatures^6–8^.

Parthenocarpy, seedless fruit formation without fertilization, is an attractive trait in fruit crops. It can offer improved fruit set and substitute for the effort of artificial pollination, even under adverse conditions. In addition, it can provide specific benefits to the tomato industry, such as the production of paste and juice by facilitating the seed elimination process. Parthenocarpy can be induced by increasing the gibberellin and auxin contents in the ovary, i.e., artificial treatment with gibberellins (GAs) and auxins^9–11^, increased GA content in mutants^12^, and an increase in the indole-3-acetic acid (IAA) content by the *iaaM* gene^13–15^.

In auxin-related signal transduction during fruit initiation, a complex of auxin/indole-3-acetic acid (Aux/IAA) and auxin response factor (ARF) protein prevents the expression of auxin-responsive genes. After fertilization, the ovule induces increases in auxin synthesis. Auxin then leads to the degradation of Aux/IAA proteins through the ubiquitin-proteasome pathway^16^. The inactivation of the Aux/IAA function results in the expression of auxin-responsive genes, followed by fruit initiation. In fact, the downregulation of the *Aux/IAA* transcription factor *SlIAA9* activates parthenocarpic fruit set in Micro-Tom^17,18^ as well as other tomato cultivars^19,20^, although the ubiquitous inhibition of *SlIAA9* function also causes abnormal phenotypes among vegetative tissues.

The promoter is usually the upstream region of a gene, and it controls where and when the gene is expressed. In genetically modified dicot plants, ubiquitous promoters such as the 35 S promoter from cauliflower mosaic virus (P35S) are widely used^21^. However, when ubiquitous promoters are used for the expression of pleiotropic genes such as *SlIAA9* or *SlDELLA*, they also cause unwanted phenotypes^18,22^. To overcome the disadvantages of these ubiquitous promoters, in recent years, many studies have reported the use of tissue-specific promoters in plant parts such as the seeds^23–25^, trichomes^26,27^, tubers^28^, roots^29,30^, pollen^31^, and petals^32^. However, research on promoters that drive the spatial and temporal expression of genes in reproductive tissues, especially in the ovary, are still rare.

Some researchers have previously reported the development of parthenocarpic tomatoes using a tissue-specific promoter and auxin biosynthesis gene combination in a heterologous system^13,15^. In research on a transgene-harboring construct consisting of an *iaaM* gene (which is involved in the biosynthesis of indole-3-acetic acid) from *Pseudomonas syringae* pv. *savastanoi* and the placental/ovule-specific *DefH9* promoter from *Antirrhinum majus*, parthenocarpy was exhibited in transgenic tomatoes.

The objective of this study was to engineer parthenocarpic tomato plants without pleiotropic effects on vegetative tissues. To do so, we developed new, flower-specific promoters from tomatoes, with a special focus on the ovary tissues. Our transgenic plant lines created with novel, specific promoters and a *SlIAA9-*RNA interference (RNAi) construct showed a vegetative phenotype similar to that of the wild type, and they also successfully formed parthenocarpic fruits.

## Results and Discussion

### Expression of *Solyc03g007780* and *Solyc02g067760* in various tissues

The first objective of our study was to develop an ovary-specific promoter. To identify the tissue-specific genes expressed in ovary tissues but not in vegetative tissues, we used RNA-seq data from 27 tissues of the tomato dwarf cultivar ‘Micro-Tom’ provided in a previous report^33^. Among the 505 genes specifically detected in the ovary samples, twenty-nine genes were selected and identified by comparison with an EST library representation of the Sol Genomics Network. *Solyc03g007780* and *Solyc02g067760* were selected by quantitative reverse transcription (qRT)-PCR analysis for further study.

**Figure 1:**
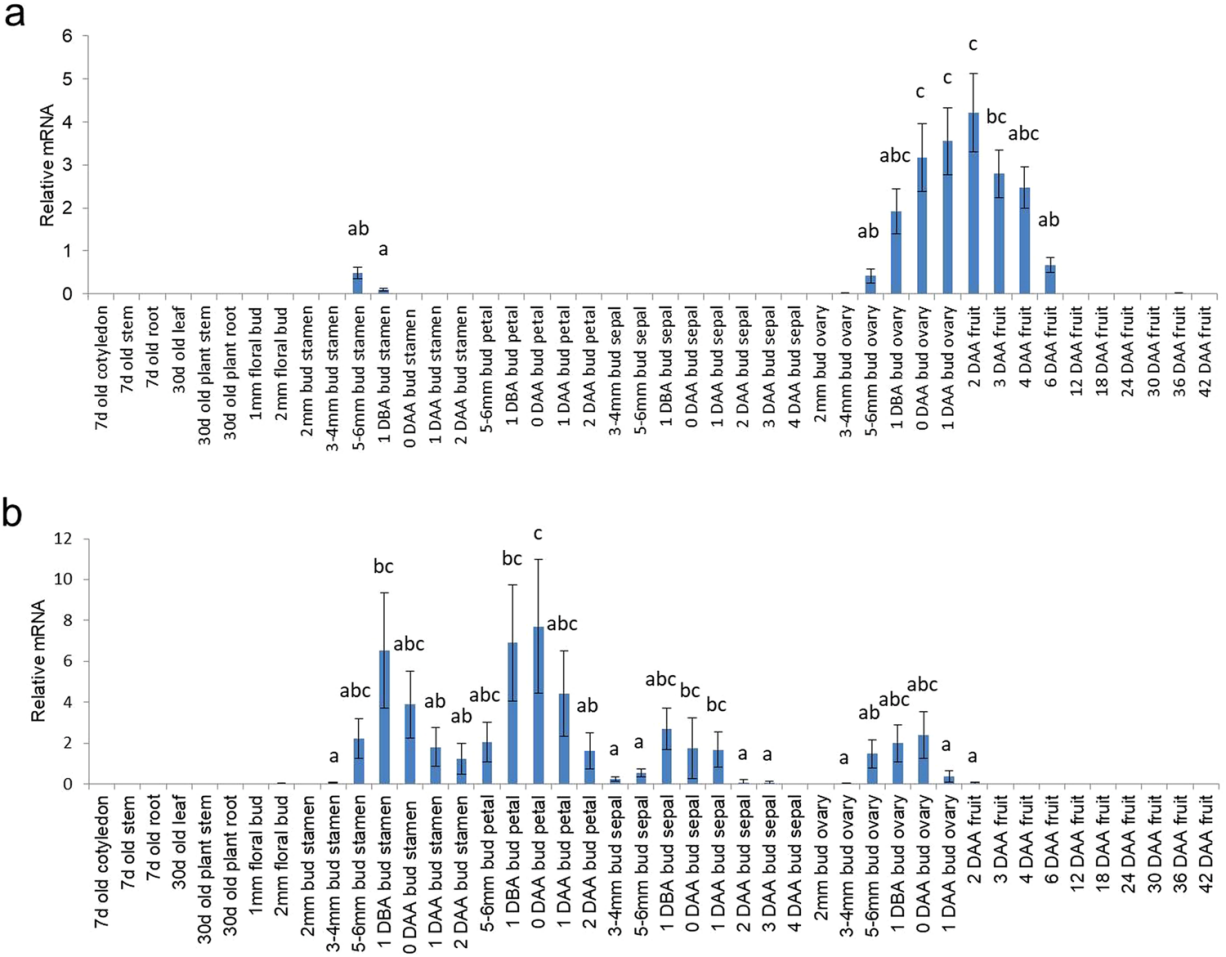
Expression of two candidate genes for promoter development. Relative mRNA levels of (**a**) *Solyc03g007780* and (**b**) *Solyc02g067760*. Values are means ± SDs of three biological replicates. Different letters denote significant differences according to Tukey’s HSD test at p < 0.01.

*Solyc03g007780* was not expressed in vegetative tissues such as the leaf, stem, and root of 7- and 30-day-old plants, but it was expressed in the ovaries at various time points and in the stamens when they reached a length of 5–6 mm and 1 day before anthesis (DBA) (Fig. 1a). In developing ovaries and pollinated ovaries (referred to as fruits in Fig. 1), the expression of *Solyc03g007780* was initiated in 5–6 mm buds, and it gradually increased until 2 days after anthesis (DAA) and decreased from 3 DAA to 6 DAA. A previous report revealed that *Solyc03g007780*, which was designated an ovule-secreted protein (OSP), was expressed in ovules at a high level but was rarely expressed in other fruit or vegetative tissues, with the exception of fruits entering the ripening stage, in *Solanum pimpinellifolium*^34^. However, these expression data showed little difference in comparison with our data (e.g., the stamen in our data and the fruits entering the ripening stage in their data).

*Solyc02g067760* was expressed in whole-flower tissues but not in vegetative tissues in this study (Fig. 1b). In the stamens and petals, *Solyc02g067760* mRNA was detected in the 5–6 mm buds, reached its peak level 1 DBA and 0 DAA, respectively, and then decreased. Its expression in the sepals gradually increased between the 3–4 mm and 1 DBA stages of buds and decreased thereafter. In developing ovaries and pollinated ovaries, the transcription of *Solyc02g067760* initiated in the 5–6 mm buds, increased until 0 DAA, and stopped after 2 DAA.

### GUS activity of the promoters of *Solyc03g007780* and *Solyc02g067760*

To develop tissue-specific promoters based on tissue-specific expressed genes, namely, *Solyc03g007780* and *Solyc02g067760*, a 2002 bp region from the start codon of *Solyc03g007780* (Psol80) or a 1996 bp region from *Solyc02g067760* (Psol60) was amplified and cloned into β-glucuronidase (GUS) expression vectors (Supplementary Fig. S1). At least two independent T_2_ lines per construct were used for the GUS histochemical analysis, and lines harboring the same construct showed the same GUS staining pattern.

**Figure 2:**
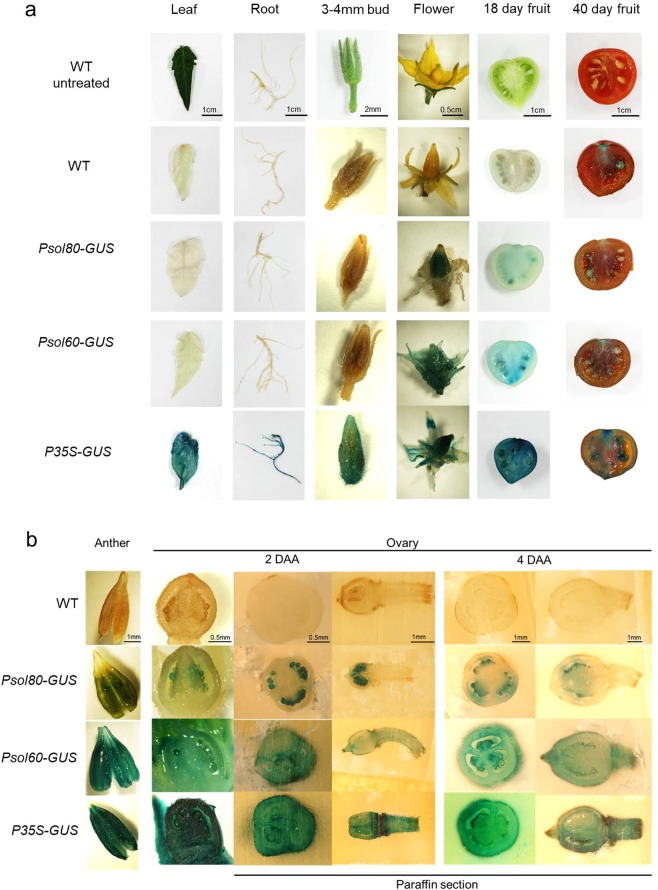
GUS histochemical assays of transgenic tomato harboring a promoter-GUS expression vector. (**a**) Images of the leaf, root, floral bud, flower, and fruit. (**b**) A stamen and paraffin-sectioned ovary under a dissecting microscope. Wild type (WT), promoter of *Solyc03g007780*-GUS line (*Psol80-GUS*), promoter of *Solyc02g067760*-GUS line (*Psol60-GUS*), and CaMV 35 S promoter-GUS (*P35S-GUS*).

In comparison, tomato plants transformed with *P35S-GUS*, the binary vector constituting the *GUS* gene under a control of the constitutive CaMV 35S promoter (P35S), showed ubiquitous GUS staining, in contrast to wild type (WT) plants, which exhibited no GUS activity (Fig. 2a). The tomato plants containing the promoter of either the *Solyc03g007780-GUS* (*Psol80-GUS*) or *Solyc02g067760-GUS* (*Psol60-GUS*) construct exhibited tissue-specific GUS staining devoid of GUS activity in the vegetative tissues. In *Psol80-GUS* lines, GUS activity was observed in anther tissue at the anthesis stage and the seeds of 18-day-old fruits (Fig. 2a). In *Psol60-GUS* lines, GUS staining was observed in the entire flower and 18-day-old fruits.

According to our qRT-PCR results, both genes were expressed in the ovaries before and after the anthesis stage (Fig. 1), followed by no expression beginning 6 DAA (*Solyc03g007780*) or 2 DAA (*Solyc02g067760*). However, in the seeds and seed-surrounding tissues of fruits in the WT as well as all promoter-GUS plants, we observed a pale blue color, despite the use of modified methods to reduce intrinsic GUS-like activity according to previous reports^35,36^. The reproductive organs of tomatoes, such as the pericarp, seed coat and embryo, have unexpected GUS-like activity^37^. Additionally, GUS protein is very stable in cells, and its half-life is up to 50 h in living cells^38^. Therefore, to reconfirm the GUS activity controlled by PSol80 and PSol60 in ovaries developing into fruits, the mRNA of the *GUS* gene was measured by qRT-PCR. In the *Psol80/60-GUS* lines, the *GUS* gene was expressed in the 0 DAA floral buds and not detected in the leaf, 3–4 mm bud, 6 DAA fruit, 18 DAA green fruit, or 40 DAA red fruit (Supplementary Fig. S2a). Although the expression level of the *GUS* gene of the *Psol80/60-GUS* lines was relatively low compared with that of the *P35S-GUS* lines, the expression patterns corresponded to those of *Solyc03g007780* and *Solyc02g067760* (Fig. 1). For more detailed observations, the sectioned ovaries at 2 and 4 DAA and the anthers at anthesis were subjected to GUS staining. In contrast to the results for the WT, tissue-specific GUS staining was observed in the *Psol80/60-GUS* lines (Fig. 2b). In the *Psol80-GUS* lines, GUS staining was observed in pollen, ovules, and a small part of the placenta and endocarp surrounding the ovule. In the *Psol60-GUS* lines, GUS activity was detected in anthers, pollen, and ovaries. The mRNA of *GUS* was also detected in the 0 DAA ovaries from *P35S-GUS* and *Psol80/60-GUS* lines but not in those from the WT (Supplementary Fig. S2b). The results of the promoter GUS assay, taken together, demonstrated that both Psol80 and Psol60 induced tissue- and developmental stage-specific expression before and after anthesis.

### Development of parthenocarpic transgenic tomato plants

The downregulation or loss of function of the *Aux/IAA* transcription factor *SlIAA9*, a negative regulator of the auxin response, resulted in the development of parthenocarpic fruits^17,18^. However, due to the ubiquitous role of *SlIAA9*, the constitutive knockdown or knockout of this gene also results in abnormal vegetative phenotypes, such as fused leaves with simple lobes. To obtain parthenocarpic tomato plants without the pleiotropic effect on vegetative tissues, Psol80 or Psol60 was used to drive the ovary-specific transcription of the RNAi constructs for *SlIAA9* knockdown (Supplementary Fig. S1). To prevent off-target effects on other Aux/IAA genes, identical *SlIAA9* mRNA region that verified targeting accuracy in a previous report^18^ was selected through further confirmation by the BLAST algorithm of the Sol Genomics Network. Through Agrobacterium-mediated transformation into ‘Micro-Tom’ cultivars, three independent RNAi lines (lines 4–17, 4–23, and 4–25) for *SlIAA9* downregulation by Psol80 (*Psol80-SlIAA9i*) and two lines (lines 6–10 and 6–18) for *SlIAA9* downregulation by Psol60 (*Psol60-SlIAA9*i) were obtained.

**Figure 3:**
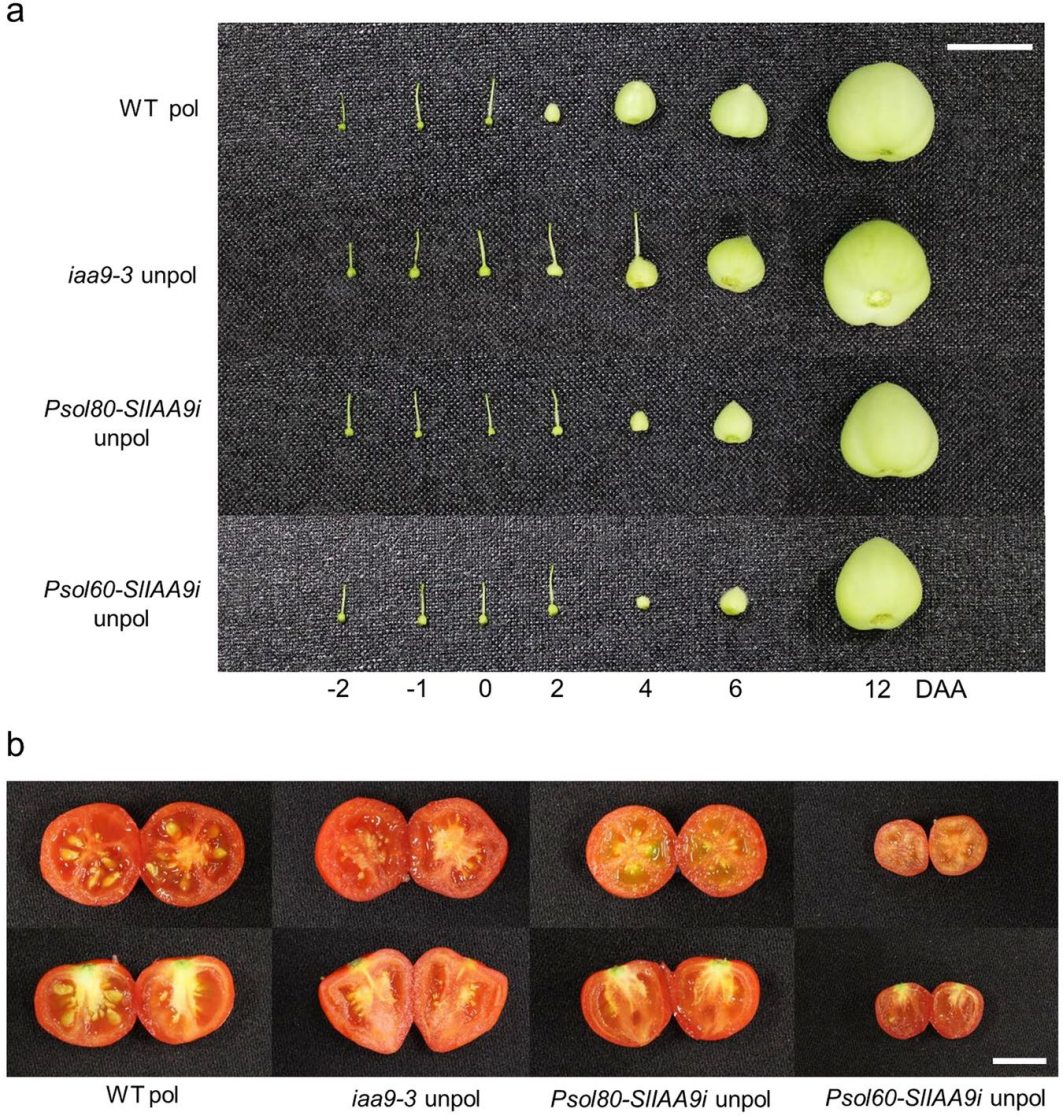
Representative fruit formation of transgenic lines. (**a**) Ovaries during early fruit development. (**b**) Mature red fruit. Pollinated wild type (WT pol), *iaa9-3* mutant (*iaa9-3*), transgenic tomato line with an *SlIAA9-*RNA interference construct controlled by the *Solyc03g007780* promoter (*Psol80-SlIAA9i)* and *Solyc02g067760* promoter (*Psol60-SlIAA9i*). Bar = 1 cm.

First, we evaluated parthenocarpic fruit formation after emasculation. In our experiment, growing ovary followed by color change from green to reddish was considered as a fruit. Both the *Psol80-SlIAA9i* and *Psol60-SlIAA9i* lines successfully formed parthenocarpic fruits, similar to the *iaa9-3* line^17^, *SlIAA9* mutant with malfunction of SlIAA9 protein, used as a control line with parthenocarpy (Fig. 3). On the other hand, most emasculated flowers of the WT withered and fell within 2 weeks without developing into fruits. Only very few unpollinated WT ovaries (approximately 2% of the unpollinated ovaries) developed into abnormal fruits with an orange color, spongy pericarp, and weight under 0.3 g. The percentage of parthenocarpic fruit formation was 63% in the *iaa9-3* mutant and ranged from 62 to 68% in the *Psol80-SlIAA9i* lines (4–17, 23, and 25) (Table 1). The *Psol60-SlIAA9i* lines showed relatively low parthenocarpic efficiency, measuring 40% (6–10) and 44% (6–18), compared with the efficiency of other tested lines. When two flowers per flower truss were emasculated, the weight of parthenocarpic fruits from the *iaa9-3* mutant was similar to that of fruits from pollinated ovaries of the WT, whereas the weight of the parthenocarpic fruits of the *Psol80-SlIAA9i* lines was up to 45% of that of the pollinated fruits of the WT. Furthermore, the parthenocarpic fruits of the *Psol60-SlIAA9i* lines exhibited a much lower weight than the pollinated fruits of the WT (up to approximately 20%).

**Table 1:** Efficiency of fruit set, weights of mature fruits, seed numbers, and yield.

	Lines	Fruit set (%; No. of fruits/flower)	Weight (g)^†^	Yield/plant^‡c^ (g)	
Emasculation^a^	WT pol	95 (97/102)	2.17 ± 0.95	36.6 ± 7.92^c^	
	WT unpol	2 (3/134)	—	—	
	4–17 unpol	66 (126/191)	0.78 ± 0.56 *	24.5 ± 4.84^b^	
	4–23 unpol	62 (114/183)	0.85 ± 0.45 *	25.3 ± 3.73^b^	
	4–25 unpol	68 (144/213)	0.97 ± 0.54 *	27.8 ± 3.07^bc^	
	6–10 unpol	40 (40/101)	0.38 ± 0.17 *	6.7 ± 4.69^a^	
	6–18 unpol	44 (70/158)	0.43 ± 0.19 *	8.7 ± 2.12^a^	
	iaa9–3 unpol	63 (79/126)	2.01 ± 1.23	26.8 ± 3.61^bc^	
Pollination^b^	**Lines**	**Fruit set (%; No. of fruits/flower)**	**Weight (g)**^**†**^	**Seed set (%)**	**No. of seeds/fruit (n = 12)**^**†**^
	WT pol	96 (57/59)	3.56 ± 0.81	100 (57/57)	38.0 ± 9.77
	4–17 pol	96 (65/68)	3.51 ± 0.79	100 (65/65)	31.9 ± 12.26
	4–23 pol	95 (62/65)	3.46 ± 0.80	98 (61/62)	37.9 ± 11.03
	4–25 pol	94 (51/54)	3.31 ± 0.83	100 (51/51)	33.9 ± 11.12
	6–10 pol	94 (50/53)	3.67 ± 0.82	100 (50/50)	37.0 ± 9.77
	6–18 pol	95 (55/58)	3.32 ± 0.96	100 (55/55)	33.6 ± 7.73
	iaa9-3 pol	58 (55/95)	3.35 ± 1.57	67 (37/55)	24.8 ± 9.02*

^a^Two flowers per flower truss were emasculated.

^b^Six flowers per plant were artificially pollinated.

^c^All the flowers from the 4th flower truss were pollinated in the WT or emasculated in *Psol80/60-*

*SlIAA9i* and the *iaa9-3* mutant for 4 weeks.

^†^Each data point represents the mean ± standard deviation.

*Asterisks indicate significant differences relative to WT pol (*P < 0.05; Student’s t-test).

- Not measured.

^‡^Each data point represents the mean ± standard deviation (n = 4–6 plants). Different letters denote significant differences between factors according to Tukey’s HSD test at p < 0.01.

Previous researchers revealed that the pollen and ovules of *SlIAA9*-downregulated lines were fertile, although a small percentage of pollinated flowers developed into seeded fruits (35%) when an *SlIAA9*-downregulated line was used as the female recipient^18^. During self-pollination of the *iaa9-3* mutant, we also observed that some fruits from pollinated ovaries did not have any seeds despite several rounds of artificial pollination. To determine whether parthenocarpic fruit set in *Psol80/60-SlIAA9i* lines also has an effect on seeded fruit formation, we measured the efficiency of fruit formation and fruit weight using six self-pollinated flowers per plant. While most of the pollinated ovaries of the WT (95%) developed into seeded fruits, i.e., fruits resulting from pollination, the *iaa9-3* mutant produced a small number of seeded fruits (67%) with fewer seeds than the pollinated fruits of the WT (Table 1). However, the *Psol80/60-SlIAA9i* lines showed efficiencies of seeded fruit formation and seed number per fruit that were similar to those of the WT. These results indicated that the *Psol80-SlIAA9i* and *Psol60-SlIAA9i* lines showed facultative parthenocarpy, in contrast to the *iaa9-3* mutant. Previous researchers reported that the early pollinated fruits of *SlIAA9*-downregulated lines were larger than those of the WT^39^. However, the weight of pollinated mature fruits from *SlIAA9-*inhibited lines (*Psol80/60-SlIAA9i* lines and *iaa9-3* mutant) was similar to that of pollinated fruits of the WT (Table 1).

### Engineered parthenocarpic tomato plants exhibit a WT-like vegetative phenotype

**Figure 4:**
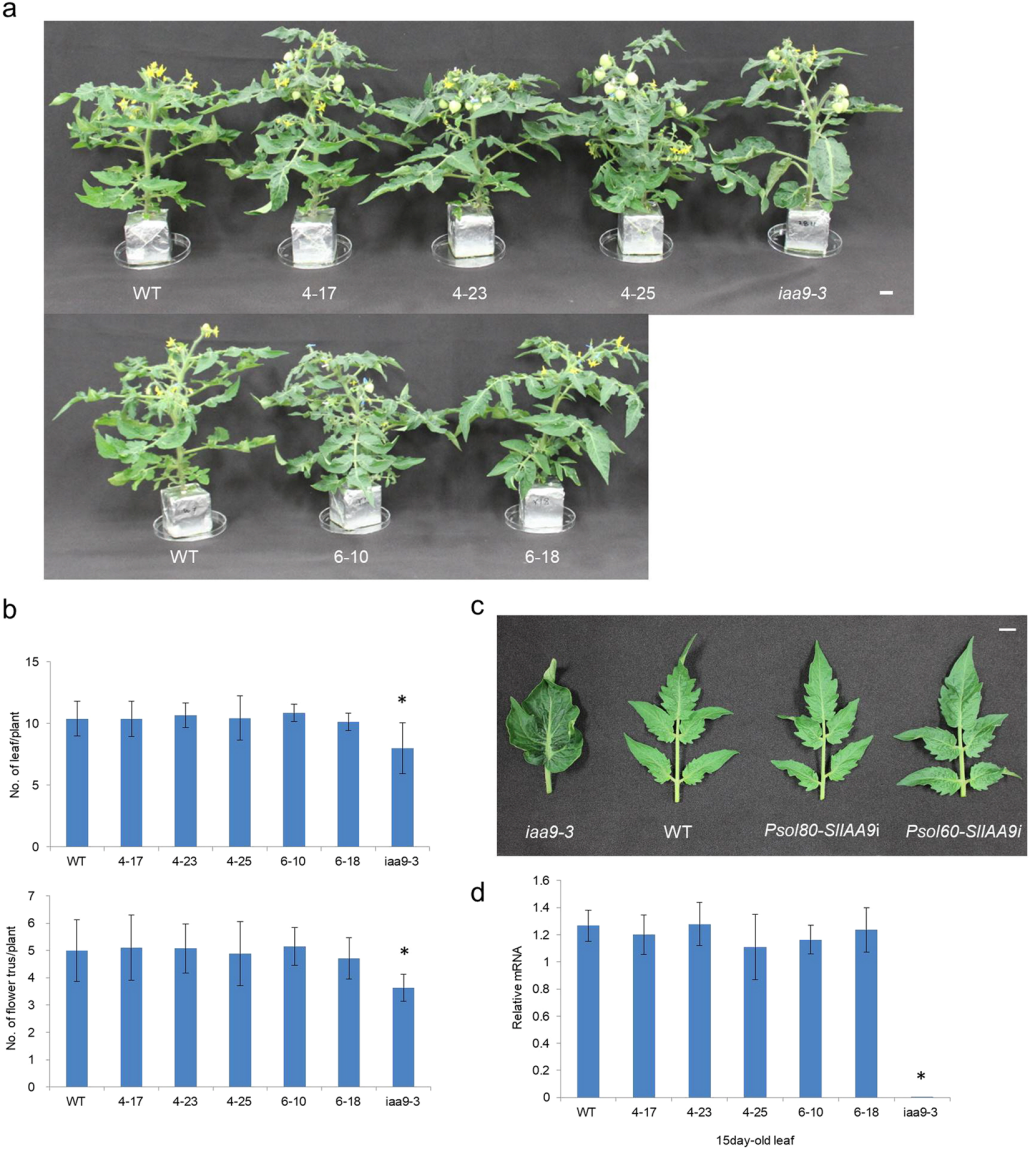
Vegetative phenotype of transgenic lines. (**a**) Plant form of 50-day-old plants. (**b**) Nos. of compound leaves and flower trusses. Values are means ± SDs of 9 plants. Asterisks indicate significant differences from the WT (P < 0.05; Student’s t-test). (**c**) Comparison of leaf morphologies. (**d**) Expression levels of *SlIAA9* in expanding leaves (15 days old). The values are the means ± SDs of 5 biological replicates. Wild type (WT), *iaa9-3* mutant (*iaa9-3*), *Psol80-SlIAA9i* lines (4–17, 23 and 25), and *Psol60-SlIAA9i* lines (6–10 and 18).

In a previous report, the inhibition of *SlIAA9* also caused altered vegetative tissue morphology, such as fused leaves, long internodes and increased lateral shoot numbers, corresponding to ubiquitous expression of *SlIAA9*^17,18^. In this study, while the *iaa9-3* mutant showed an abnormal plant form with a sparse leaf arrangement, the *Psol80/60-SlIAA9i* lines (4–17, 4–23, 4–25, 6–10, and 6–18) presented plant forms similar to those of the WT (Fig. 4a). To present the difference in plant form numerically, we counted the number of compound leaves and flower trusses on 50-day-old plants. All *Psol80-SlIAA9i* and *Psol60-SlIAA9i* lines formed similar numbers of compound leaves and flower trusses compared to those of WT (Fig. 4b). However, the *iaa9-3* mutant formed significantly fewer flower trusses and compound leaves. In addition, the WT and all *Psol80/60-SlIAA9i* lines exhibited pinnately compound leaves with lobed leaflets, whereas the *iaa9-3* mutant formed fused leaves with simple lobes (Fig. 4c). Because the downregulated level of *SlIAA9* in leaves showed a positive correlation with the severity of altered leaf morphology^18^, we also examined the mRNA level of *SlIAA9* in leaves to explain the normal leaf morphology of the *Psol80/60-SlIAA9i* lines. Consistent with the WT-like leaf phenotype, the *Psol80/60-SlIAA9i* lines showed *SlIAA9* mRNA levels similar to those of the WT, in contrast to the *iaa9-3* mutant, which presented no intact *SlIAA9* mRNA in leaves (Fig. 4d). Furthermore, *Solyc03g007780* and *Solyc02g067760* expression was also not detected in the expanding leaves of the corresponding transgenic lines (Supplementary Fig. S3). These results suggested that the mRNA level of *SlIAA9* was not affected by *SlIAA9*i in leaves of the *Psol80/60-SlIAA9i* lines.

### Tissue specific downregulation of *SlIAA9* is responsible for parthenocarpic fruit formation in *Psol80/60-SlIAA9i* lines

**Figure 5:**
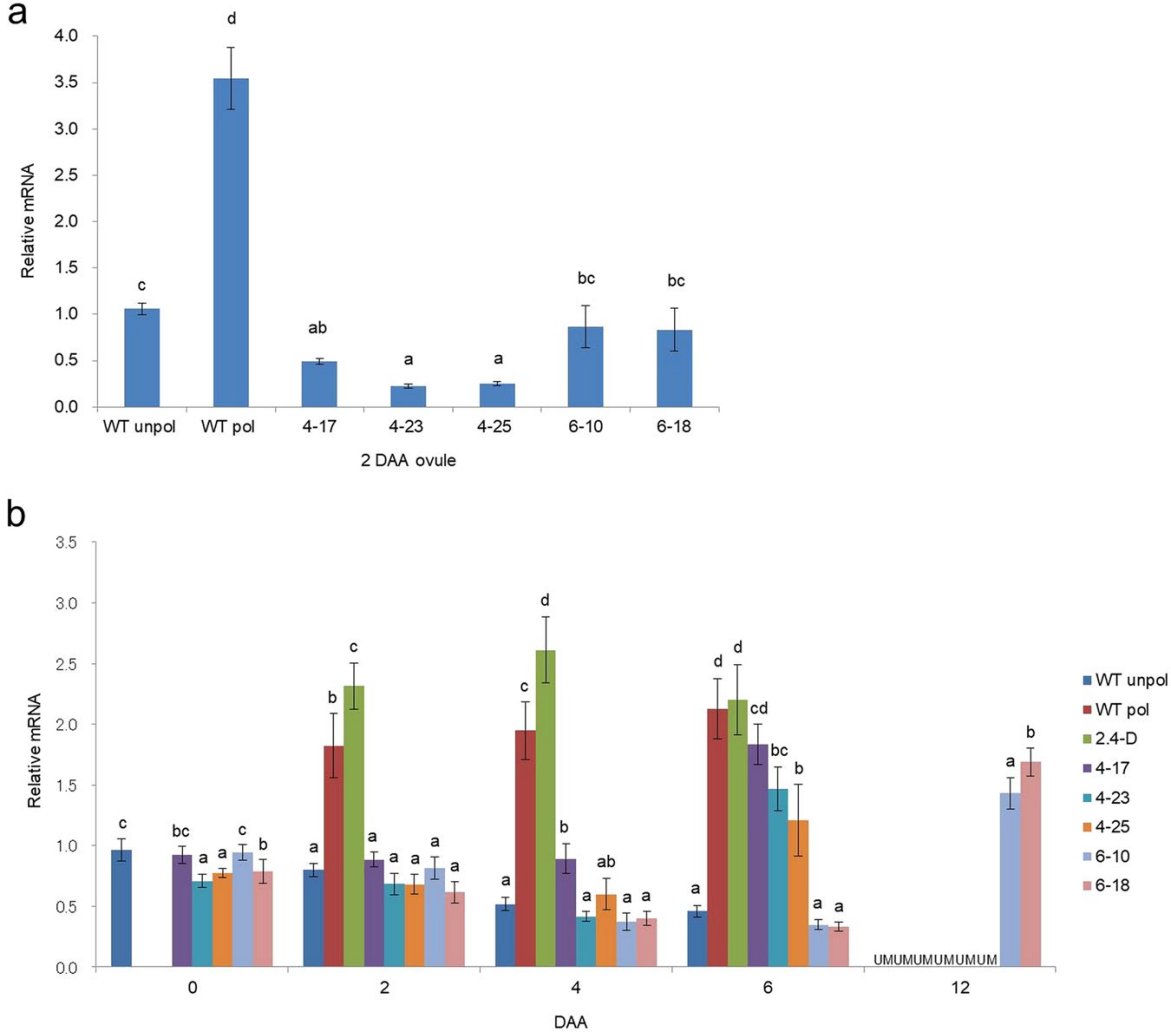
Expression levels of *SlIAA9*. (**a**) Ovules 2 DAA. (**b**) Ovaries 0, 2, 4, 6 and 12 days after anthesis (DAA). Unpollinated (WT unpol), pollinated (WT pol), and 2,4-dichlorophenoxyacetic acid-treated (2,4-D) ovaries of the wild type. Unpollinated ovaries of *Psol80-SlIAA9i* lines (4–17, 23 and 25), *Psol60-SlIAA9i* lines (6–13 and 18), and the *iaa9-3* mutant (iaa9-3). Values are means ± SDs from three biological replicates. Different letters denote significant differences according to Tukey’s HSD test at p < 0.01 at each time point. UM indicates unmeasured.

Knockdown of *SlIAA9* mRNA mimics increased auxin followed by subsequent parthenocarpic fruit formation^16,18^. In unpollinated ovaries, *SlIAA9* mRNA was more strongly distributed in the ovule and placenta than in other ovary tissues^39^, implying that the transcript level of *SlIAA9* in those tissues is important for parthenocarpic fruit set by *SlIAA9* inhibition. Consistent with this interpretation, increased IAA in the placental/ovule tissue was sufficient for induction of parthenocarpic fruit set^14,15^. In the present study, Psol80 and Psol60 also induce the expression of the genes in ovules and placenta (Fig. 2b). Therefore, we assessed the mRNA level of *SlIAA9* in ovules of *Psol80/60-SlIAA9i* lines. The level of *SlIAA9* mRNA was significantly or slightly lower in the ovules of the *Psol80-SlIAA9i* lines and *Psol60-SlIAA9i* lines, respectively, than in the ovules of the unpollinated or pollinated WT (Fig. 5a). This result suggested that the parthenocarpic fruit formation was induced by downregulation of *SlIAA9* mRNA -in the *Psol80/60-SlIAA9i* lines.

In the assay of *SlIAA9* transcript levels in ovaries, the transcript level of *SlIAA9* in unpollinated ovaries of the WT continuously decreased between 0 and 6 DAA without ovary growth (Fig. 5b). However, the level dramatically increased 2 DAA and remained at that level with ovary development into fruit by pollination or 2,4-D treatment. The level of *SlIAA9* mRNA in developing ovaries of the *Psol80/60-SlIAA9i* lines was not increased compared with that of unpollinated ovaries of the WT between 0 and 4 DAA, but it rapidly increased 6 DAA in the *Psol80-SlIAA9i* lines or 12 DAA in the *Psol60-SlIAA9i* lines.

Surprisingly, an obvious decrease in *SlIAA9* mRNA level by *SlIAA9* RNAi in ovaries of the *Psol80/60-SlIAA9i* lines was not observed. To describe the insufficient downregulation of *SlIAA9* in ovaries, the activity of Psol80/60 had to be considered. As shown in Fig. 2b and Supplementary Fig. S2b, Psol80 induced the transcription of the gene in the ovule and adjacent tissues, which occupied a small portion of the entire ovary (containing the columella, ovary wall, placenta, and ovule), resulting in low expression in the entire ovary. Psol60 also conferred much lower expression to the controlled gene compared with P35S. These results implied that the distribution or the amount of *SlIAA9* dsRNA expressed by Psol80/60 was not sufficient to cause a remarkable decrease in *SlIAA9 mRNA* level in the entire developing ovary. Meanwhile, despite the development of ovaries into fruits in the *Psol80/60-SlIAA9i* lines (Fig. 3A), the increase in *SlIAA9* mRNA level was delayed until 6 DAA in the *Psol80-SlIAA9i* lines and 12 DAA in the *Psol60-SlIAA9i* lines. These expression patterns showed an inverse correlation with the expression pattern of *Solyc03g007780* in the pollinated and unpollinated ovaries of WT and *Solyc02g067760* in the unpollinated ovaries of WT, reflecting the activity of each promoter (Fig. 1, Supplementary Fig. S4). *Solyc03g007780* was expressed considerably from 0 DAA to 4 DAA, and at the same time, the *SlIAA9* mRNA level was not increased. However, at the time point when the expression of *Solyc03g007780* was extremely reduced (6 DAA), the transcripts of *SlIAA9* mRNA increased. The same expression pattern was observed in ovaries 12 DAA in the *Psol60-SlIAA9i* lines. These results suggest that the increase of *SlIAA9* mRNA by ovary development was partially suppressed during RNAi constructs for *SlIAA9* knockdown expressed by two promoters.

According to a previous report, the percentage of parthenocarpy in *SlIAA9*-downregulated tomato lines was negatively correlated with the level of *SlIAA9* mRNA^18^. In our research, we also observed that parthenocarpic lines with lower levels of *SlIAA9* mRNA in ovules showed a higher efficiency of parthenocarpy (Table 1, Fig. 5a). In addition, we found a negative correlation between the transcript level of *SlIAA9* in ovules and the weight of the mature fruits; i.e., strongly downregulated lines showed larger fruit formation (Table 1, Fig. 5a,).

### Fruit quality and yield of parthenocarpic fruits in *Psol80/60-SlIAA9i* lines

We also examined the fruit quality and yield of parthenocarpic fruits of the *SlIAA9*-inhibited lines. There was no significant difference in pH between the parthenocarpic fruits of *SlIAA9*-inhibited lines and the pollinated fruits of the WT. However, in the titratable acidity (TA) assay, parthenocarpic fruits showed increased TA compared with pollinated fruits of the WT (Table 2). While the parthenocarpic fruits of the *Psol80-SlIAA9i* lines and *iaa9-3* mutant presented slightly increased TA, those of the *Psol60-SlIAA9i* lines had much higher TA. Although the value of TA is mainly determined by concentrations of citric acid, malic acid, and phosphate in tomato fruits, phosphate concentrations showed no difference with between cultivars with various TA^40^. Therefore, the higher TA suggests that the parthenocarpic fruits had high acid concentrations compared with that in the pollinated fruits of the WT.

**Table 2:** Total soluble solids (TSS, °Brix), titratable acidity (TA), and β-carotene and lycopene contents in mature fruits.

Lines	pH	TA (%)	TSS	β-carotene (μg/g)	Lycopene (μg/g)
WT pol	3.76 ± 0.05^ab^	0.079 ± 0.0073^a^	5.6 ± 0.12^a^	26.26 ± 3.01^a^	90.68 ± 5.14^a^
4–17	3.66 ± 0.10^a^	0.099 ± 0.0074^bc^	7.8 ± 0.55^b^	25.16 ± 2.35^a^	89.83 ± 3.99^a^
4–23	3.80 ± 0.11^ab^	0.091 ± 0.0083^ab^	7.2 ± 0.92^b^	21.86 ± 1.87^a^	87.61 ± 5.02^a^
4–25	3.72 ± 0.07^ab^	0.088 ± 0.0077^ab^	6.8 ± 0.80^ab^	34.87 ± 4.99^a^	99.41 ± 2.94^a^
6–10	3.72 ± 0.04^ab^	0.115 ± 0.0087^c^	9.9 ± 0.62^c^	34.40 ± 17.23^a^	89.72 ± 18.03^a^
6–18	3.70 ± 0.01^ab^	0.116 ± 0.0082^c^	10.0 ± 0.42^c^	27.13 ± 7.00^a^	92.80 ± 5.54^a^
*iaa9-3*	3.84 ± 0.05^b^	0.084 ± 0.0043^ab^	6.9 ± 0.19^ab^	39.07 ± 8.36^a^	98.37 ± 6.27^a^

Each data point represents the mean ± standard deviation (n = 5). Different letters denote significant differences between factors according to Tukey’s HSD test at p < 0.01.

Parthenocarpic tomato fruits induced by increased IAA contents in the ovules have similar fruit weights, slightly increased levels of total soluble solids (TSS) compared with those of pollinated fruits of the WT^13^. The parthenocarpic fruits of *iaa9-3* mutant also showed higher levels of total soluble solids (TSS) than the fruits of the WT (Table 2). In particular, low-weight parthenocarpic fruits from the *Psol80/60-SlIAA9i* lines exhibited much higher TSS levels than pollinated fruits of the WT. Generally, the *Psol80/60-SlIAA9i* lines formed lower-weight parthenocarpic fruits than the *iaa-3* mutant line, and the fruits of these lines had higher TA and TSS values. This result also suggested that low-weight parthenocarpic fruits had higher TA and TSS values.

In the evaluation of fruit color, the parthenocarpic fruits of the *iaa9-3* mutant and *Psol80/60-SlIAA9i* lines presented higher chroma and hue values, indicating more color saturation and a redder color than observed for the pollinated WT fruits^41^ (Table 3). The more saturated and redder color of parthenocarpic fruits in the *Psol80/60-SlIAA9i* lines also suggested that the fruits of these lines were induced by successful *SlIAA9* inhibition, similar to the case of parthenocarpic fruits in the *iaa9-3* mutant. In addition, since lycopene is the main carotenoid responsible for the red color of tomato fruits^42^, we also measured the lycopene and β-carotene contents in the parthenocarpic fruits of *SlIAA9*-inhibited lines (Table 2). However, there was no difference in β-carotene or lycopene content compared with the content in pollinated fruits from the WT. Additionally, the L* values, which express the lightness of fruits and have positive correlations with total carotenoid and lycopene contents^43^, did not show significant differences between the WT and *SlIAA9*-inhibited lines (Table 3). These results suggested that the saturated red color of parthenocarpic fruits in the *SlIAA9-*inhibited line was caused by other pigments, not a high lycopene content.

**Table 3:** Color index (L*, a*, chroma, and hue) of mature fruits.

Lines	Color L*	a*	Chroma	Hue
WT pol	40.1 ± 1.45^a^	43.0 ± 2.53^a^	51.3 ± 3.29^a^	0.58 ± 0.034^b^
4–17	42.0 ± 2.22^a^	53.4 ± 4.46^bc^	61.4 ± 4.24^b^	0.51 ± 0.072^ab^
4–23	40.3 ± 2.28^a^	54.0 ± 3.67^bc^	60.7 ± 4.76^b^	0.47 ± 0.030^a^
4–25	40.4 ± 1.87^a^	53.9 ± 4.14^bc^	60.3 ± 4.83^b^	0.47 ± 0.035^a^
6–10	39.3 ± 1.09^a^	52.0 ± 2.18^b^	58.2 ± 1.61^b^	0.46 ± 0.045^a^
6–18	40.2 ± 3.11^a^	54.0 ± 1.91^bc^	60.7 ± 3.86^b^	0.47 ± 0.071^a^
*iaa9-3*	40.4 ± 1.24^a^	57.0 ± 2.80^c^	63.6 ± 2.58^b^	0.45 ± 0.039^a^

Each data point represents the mean ± standard deviation (n = 20 except n = 8 of 6 lines). Different letters denote significant differences between factors according to Tukey’s HSD test at p < 0.01.

To compare the yield of parthenocarpic fruits of *Psol80/60-SlIAA9i* lines to that of pollinated fruits of the WT, all the flowers from the 4th flower truss were pollinated or emasculated for 4 weeks. The yield of the *iaa9-3* mutant was up to 73% of that of the WT, whereas that of the *Psol80-SlIAA9i* and *Psol60-SlIAA9i* lines was up to 76% and 24%, respectively, despite these lines expressing a WT-like leaf number and morphology (Table 1, Fig. 4b,c). This change in yield is thought to be the result of relatively low parthenocarpic fruit set efficiency and fruit weight due to insufficient downregulation of *SlIAA9* in the ovule.

The objective of this study was to make parthenocarpic tomato plant without abnormal vegetative phenotype. In this study, the promoters of *Solyc03g007780* and *Solyc02g067760* induced tissue specific expression of genes. Downregulation of *SlIAA9* by the two promoters presented facultative parthenocarpic fruit formation without abnormal vegetative phenotype in transgenic tomato lines.

## Methods

### Plant materials and growth conditions

The tomato (*Solanum lycopersicum* L.) dwarf cultivar ‘Micro-Tom’ was used as the background for all the plant materials used in this study. Seeds were placed on filter paper with deionized water for 7 days at 25 °C. Germinated seeds were transplanted onto rockwool (75 × 75 × 65 mm; Grodan), irrigated with a nutrient solution (Otsuka Chemical) with an electrical conductivity (EC) of 1.8 dS m^−1^, and incubated under 16 h light/8 h dark conditions with fluorescent light at 300 μmol m^−2^ s^−1^ and a temperature of 25 °C. For the preparation of unpollinated or pollinated ovaries, flower buds were emasculated one day before anthesis or pollinated at anthesis. For 2,4-D-treated ovaries, used as a parthenocarpic fruit control induced by auxin traeatment^11^, the buds were emasculated one day before anthesis, and 4 µl of 2,4-dichlorophenoxyacetic acid (2,4-D) (Sigma-Aldrich) solution containing 20 ng of 2,4-D, 5% ethanol and 0.1% Tween 20 was applied to them at anthesis. In the fruit yield test, all the flowers at the anthesis stage on 50-day-old plants were pollinated or emasculated for 4 weeks.

### DNA and RNA extraction and cDNA synthesis

Genomic DNA and total RNA were extracted using a DNeasy or RNeasy plant mini kit (Qiagen) according to the manufacturer’s instructions. Total RNA was treated with a DNA-free RNA kit (Zymo Research) to eliminate DNA contamination. First-strand cDNA was synthesized from 1 μg of total RNA using the PrimeScript II 1st strand cDNA Synthesis Kit (Takara-bio) and oligo dT primers. The synthesized cDNA was subjected to PCR to confirm genomic DNA-free cDNA with a set of SGN-U314153 (CAC) gene primers: forward, CCTCCGTTGTGATGTAACTGG, and reverse, ATTGGTGGAAAGTAACATCATCG^44^.

### Vector construction and tomato transformation

To obtain the promoter region and *SlIAA9* fragments, the genomic DNA or cDNA from the ovaries was amplified in 50 μl of PCR mixture with 5 μl of 10x buffer, 5 μl of 2 mM dNTPs, 1 U of KOD Plus Neo (Toyobo), and 1 μl of 10 μM primers (Supplementary Table S1). The thermal cycling procedure consisted of 5 min at 98 °C, 28 cycles of amplification consisting of denaturation for 30 s at 98 °C, annealing for 30 s at 60 °C, and extension for 1 min at 68 °C, and a final extension at 68 °C for 3 min. For the promoter:GUS assay, promoter amplicons with *Hind*III (5′) and *Sal*I (3′) restriction sites for *Solyc03g007780* or with *Sal*I (5′) and *Bam*HI (3′) restriction sites for *Solyc02g067760* were cloned into the pBI101-GUS vector. For the RNAi vector, an amplified promoter with *Avr*2 (5′) and *Xho*1 (3′) was inserted into the pBI-sense, antisense gateway vector (Inplanta Innovations) instead of P35S. Subsequently, 715 bp *SlIAA9* amplicons were cloned into the pCR8/GW/TOPO vector and inserted into the pBI-sense, antisense gateway vector using Gateway LR Clonase (Thermo Fisher). The vector construct used in this study is shown in Supplementary Fig. S1.

Transgenic tomato production was conducted by *Agrobacterium*-mediated transformation according to a method previously described^45^. The transgenic lines were subjected to PCR using promoter and gene-specific primer sets and a Southern blot analysis to find the homozygous-independent lines.

### Quantitative reverse transcription PCR

The expression levels of the genes were assessed by quantitative reverse transcription PCR (qRT-PCR) with a Dice Real-Time thermal cycler and SYBR Premix Ex Taq II following the manufacturer’s protocol (Takara-bio). Diluted cDNA was used as a template in 25 μl of PCR-amplification reaction mixture containing 12.5 μl of SYBR Premix Ex Taq II (Takara-bio) and 1 μl of the 10 μM primer set. A dissociation curve analysis was also performed to confirm primer compatibility. The relative expression of the genes of interest was measured by the standard curve method with three biological replicates. *SAND* was used as a reference gene^44^. The primer set used for qRT-PCR is described in Supplementary Table S2.

### GUS and histological assay

Histochemical ß-glucuronidase (GUS) analysis was performed with 5-bromo-4-chloro-3-indolyl-b-D-glucuronide (X-Gluc) using a previously described method, with a slight modification: phosphate buffer (pH of 8.0) and 20% methanol^35,36,38^. The tissues were incubated in the GUS staining solution at room temperature for 16 h, followed by washing with 70% ethanol to eliminate chlorophyll. Paraffin sectioning was performed according to previously described methods^46^.

### Fruit characteristic analysis

For fruit analysis, twenty flowers per plant were emasculated or pollinated, and six fruits per plant were maintained for the experiment. Forty-eight-day-old fruits were subjected to color and weight measurements and stored at −80 °C for further experiments. The color was rated on the middle surface of the fruits with a Minolta Color Reader CR-10 (Konicaminolta). The color parameters describing lightness indicate the range from black (0) to white (100) (L*) and the color direction on the red to green scale (a*) and on the yellow to blue scale (b*). The chroma (color saturation) was calculated as (a*^2^ + b*^2^)^1/2^. The hue (h, term used for the classification of red, yellow, blue, and green) was measured by taking the arctan(b/a).

Frozen fruits were ground into a fine power and used to measure the total soluble solids (TSS), pH, titratable acidity (TA), and β-carotene and lycopene contents. The TSS was measured using a PAL-J refractometer (Atago). The pH and TA were analyzed using 1 g of fruit powder homogenized into 10 ml of distilled water according to a previously described method^47^. Oxalic acid was used as a standard for the titrant calculation. The β-carotene and lycopene contents were measured and calculated according to a previously described method^48^. Supernatant from the extract of 300 mg of fruit powder with 3 mL of acetone/hexane (4:6, v/v) was tested for its absorption using a Beckman DU 640 UV/Vis spectrophotometer (Beckman Coulter). The β-carotene (C_CAR_) and lycopene (C_LYC_) concentrations were calculated using the following equations:

$$\begin{array}{rcl}{{\rm{C}}}_{{\rm{CAR}}} & = & 0.216({{\rm{A}}}_{663})-1.22({{\rm{A}}}_{645})\\  &  & -\,0.304({{\rm{A}}}_{505})+0.452({{\rm{A}}}_{453})\\ {{\rm{C}}}_{{\rm{LYC}}} & = & -0.04584({{\rm{A}}}_{663})+0.204({{\rm{A}}}_{645})\\  &  & +\,0.372({{\rm{A}}}_{505})-0.0806({{\rm{A}}}_{453})\end{array}$$

## Supplementary information

Supplementary information
