# Supplementary material for: Genetic engineering of parthenocarpic tomato plants using transient SlIAA9 knockdown by novel tissue-specific promoters
Source: Sci Rep. 2019 Dec 11;9:18871. doi: 10.1038/s41598-019-55400-7 (PMC6906307; doi:10.1038/s41598-019-55400-7)
Supplement: Supplementary file 1 — Supplementary information [file 41598_2019_55400_MOESM1_ESM.docx]

**Supplementary information**

**Title:**

**Genetic engineering of parthenocarpic tomato plants using transient *SlIAA9* knockdown by novel tissue-specific promoters**

Ji-Seong Kim^1^, Kentaro Ezura^1^, Jeongeun Lee^1^, Tohru Ariizumi^1,2^, Hiroshi Ezura^1,2*^

**Affiliations:**

^1^Faculty of Life and Environmental Sciences, University of Tsukuba, Tennodai 1-1-1 Tsukuba, Ibaraki 305-8572, Japan.

^2^Tsukuba Plant Innovation Research Center, University of Tsukuba, Tennodai 1-1-1, Tsukuba, Ibaraki, 305-8572, Japan

*Corresponding author

e-mail : [ezura.hiroshi.fa@u.tsukuba.ac.jp](mailto:ezura.hiroshi.fa@u.tsukuba.ac.jp)

tel: (81) 029-853-7263

**
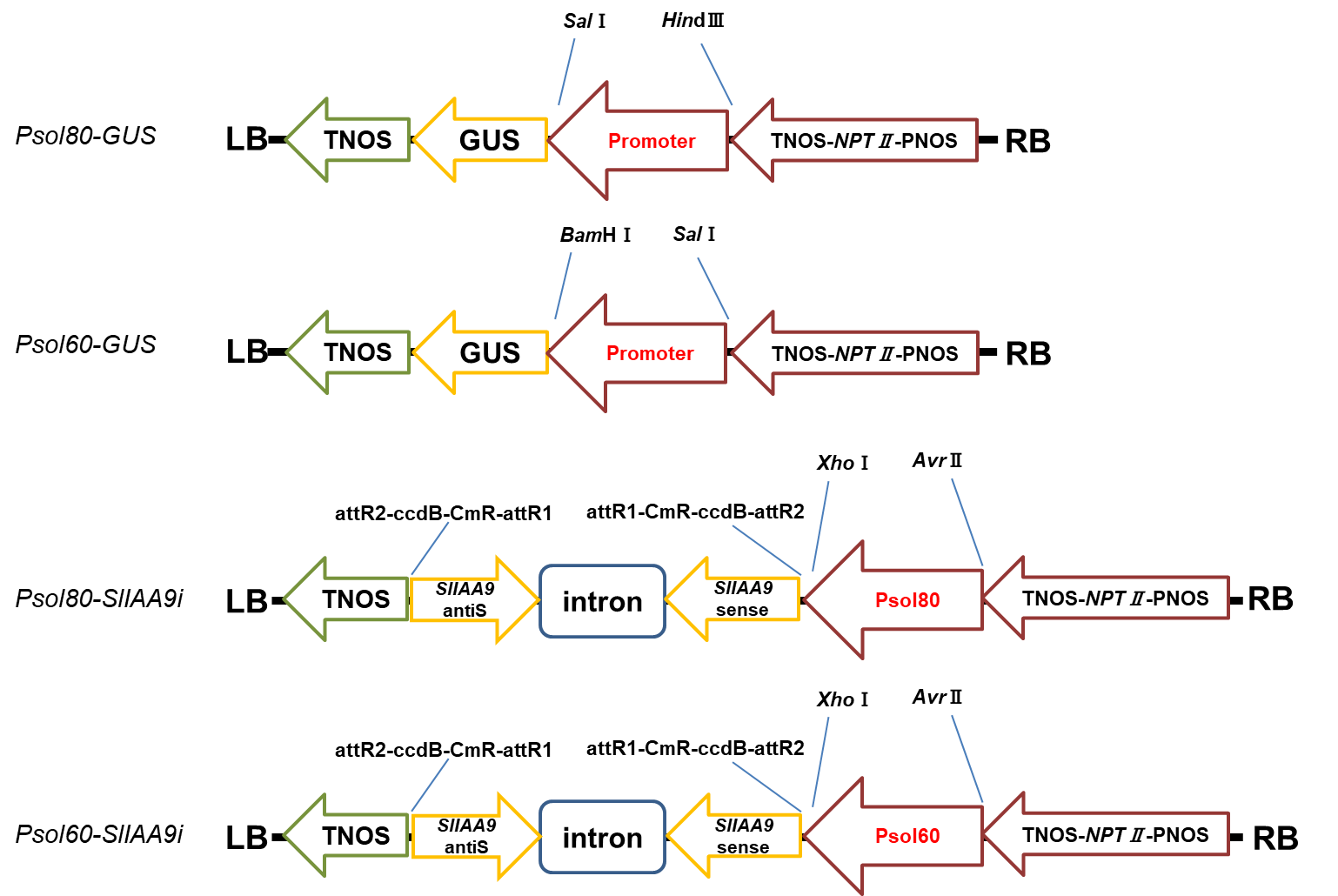
**

**Supplementary Fig. S1** Schematic representation of the T-DNA region of the vectors

**
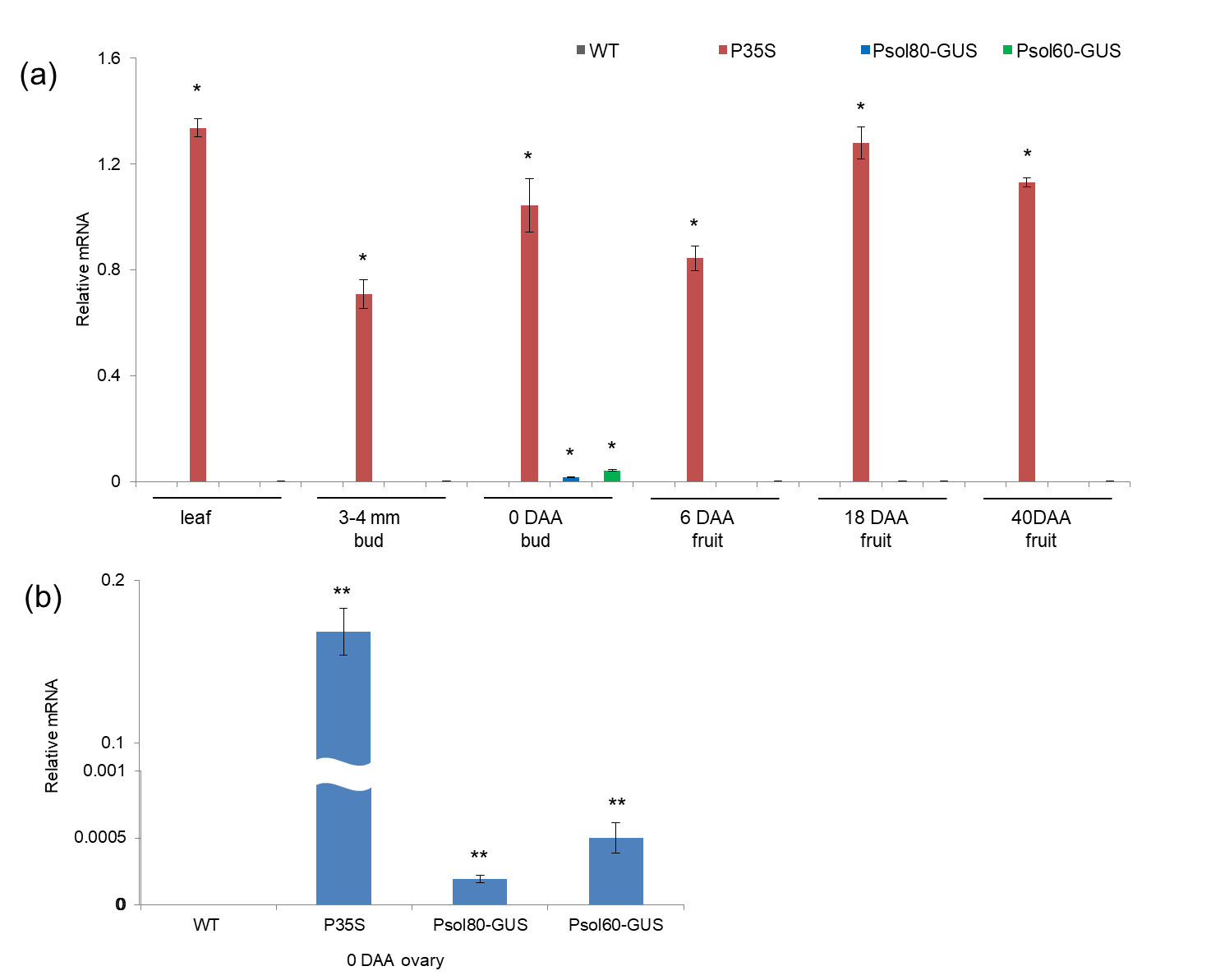
**

**Supplementary Fig. S2** Relative expression level of the *β-glucuronidase* (*GUS)* gene in transgenic plants carrying the promoter-GUS construct. (a) leaf, 3-4m bud, 0, 6, 18, and 40 DAA fruit (b)In ovary at anthesis (0DAA). Wild type (WT). P35S-GUS. Psol80-GUS plant. Psol60-GUS plant. Values are mean ± SDs of three biological replicates. Asterisks indicate signiﬁcant differences from each sample of WT (**P < 0.01, *P < 0.05; Student’s t–test)

**
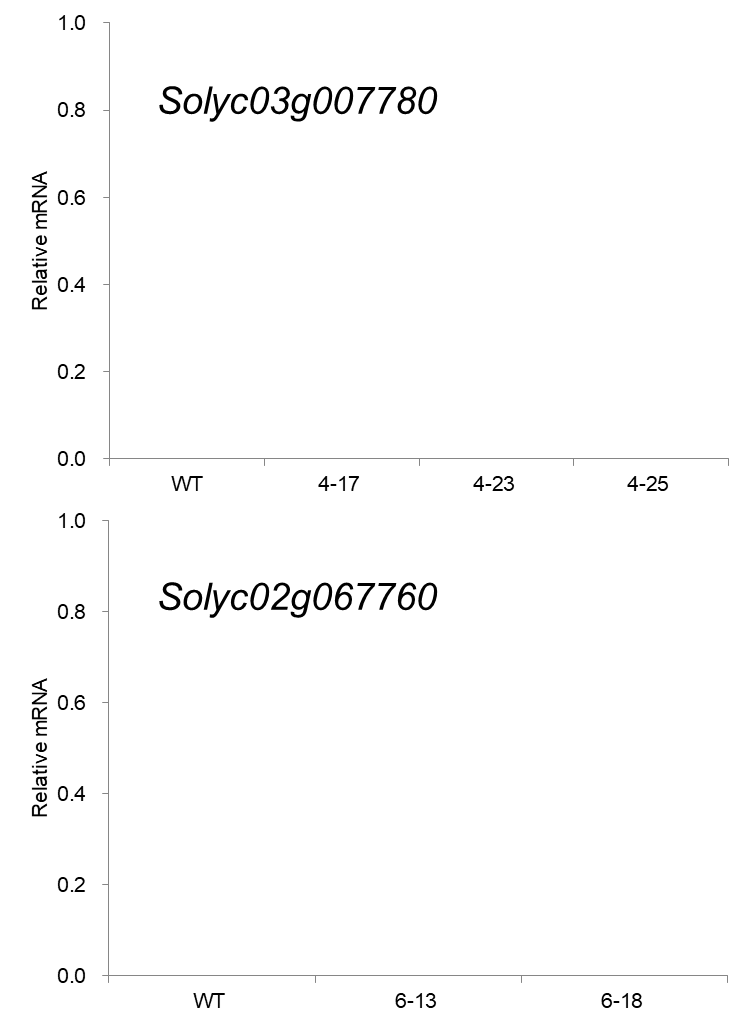
**

**Supplementary Fig. S3** Expression levels of the *Solyc03g007780* and *Solyc02g067760* genes in the expanding leaves. Values are means ± SDs of 5 biological replicates. Wild type (WT), *Psol80-SlIAA9i* lines (4-17, 23 and 25) and *Psol60-SlIAA9i* lines (6-10 and 18)

**
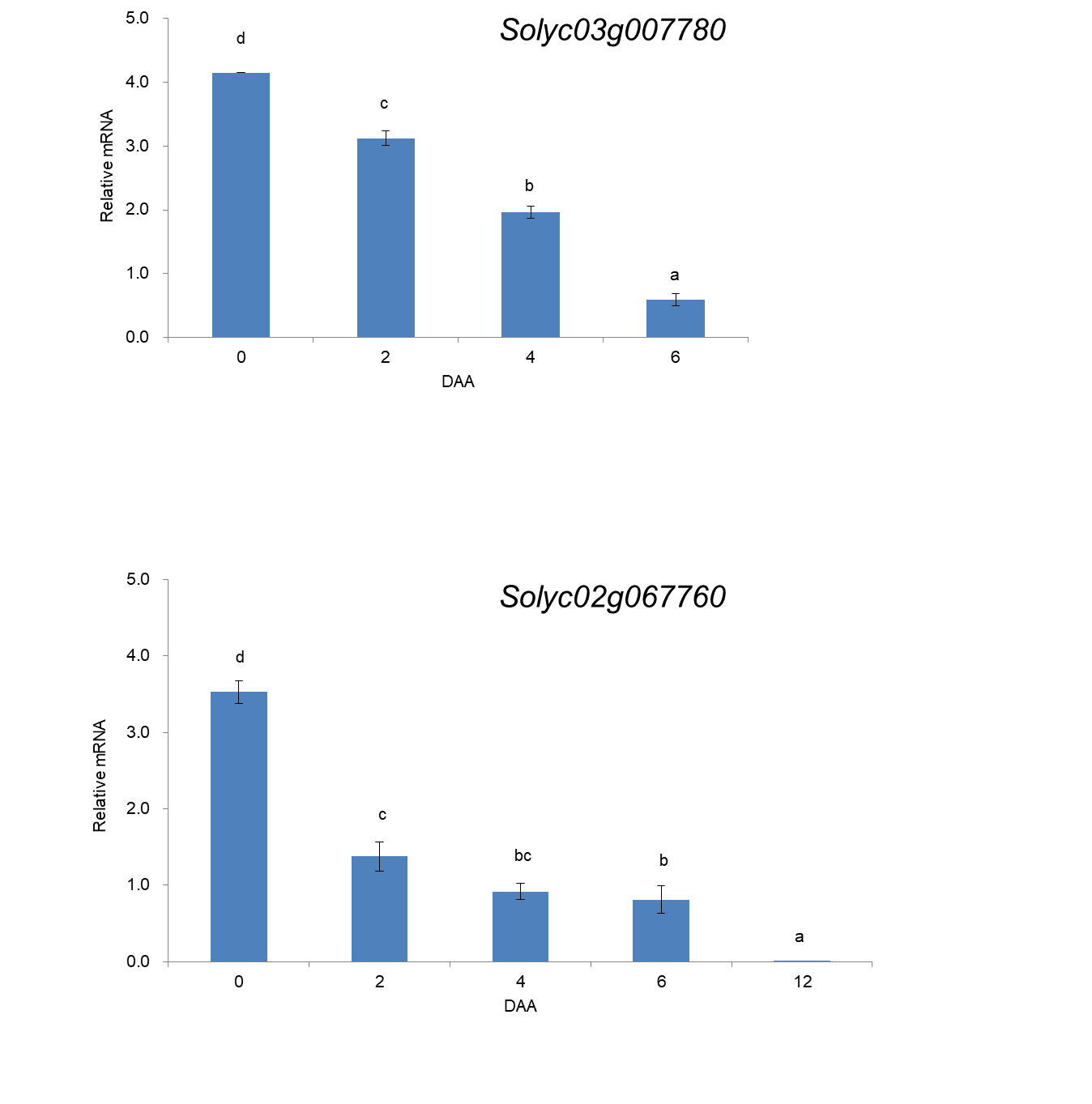
**

**Supplementary Fig. S4** Expression levels of *Solyc03g007780* and *Solyc02g067760* in unpollinated ovaries of wild type at 0, 2, 4, 6 and 12 days after anthesis (DAA). Values are means ± SD from three biological replicates. Different letters indicate significant differences according to Tukey’s HSD test at p<0.01 at each time point.

**Supplementary Table S1** List of primers used for vector construction

|  | Locus | Definition | Sequence (5’ – 3’) |
| --- | --- | --- | --- |
| Promoter | Solyc03g007780 | OSP | Forward, ACGGAGTGTGCCACATAAGAC |
|  |  |  | Reverse, AGCTTTTTTTTTTTCCCTTCTCAC |
|  | Solyc02g067760 | MYB305 | Froward, GTTCCTAGCTTTGACACACAAGAG |
|  |  |  | Reverse, GTAGAGAGAGGAAGATGAGAGA |
| RNAi | Solyc04g076850 | AUX/IAA | Forward, TGGCCACCCATTCGATCTTTTAG |
|  |  | protein 9 | Reverse, ACAAACTCCAATATCAAACGG |

**Supplementary Table S2** List of primers used for qRT-PCR

| Locus |  | Sequence (5’-3’) | Product size (bp) |
| --- | --- | --- | --- |
| Solyc03g115810 | F | TTGCTTGGAGGAACAGACG | 164 |
|  | R | GCAAACAGAACCCCTG AATC |  |
| Solyc03g007780 | F | GGATGGAGACTCTTGCTCTGAT | 130 |
|  | R | ATTAGGGCGGTTGGATGAAG |  |
| Solyc02g067760 | F | GGAGGACTAGGATTCAGAAGCAC | 238 |
|  | R | CAGATATCCTCCATGCTCCAC |  |
| Solyc04g076850 | F | CTCAGGCTCGGTCTACCTG | 196 |
|  | R | CCTCTGAGAATCCATCCATAGC |  |
| ß-glucuronidase | F | CCGGGTGAAGGTTATCTCTATG | 160 |
|  | R | CATGACGACCAAAGCCAGTA |  |
